# Supplementary figures and images for: Diurnal regulation of metabolism by Gs-alpha in hypothalamic QPLOT neurons
Source: PLoS One. 2023 May 4;18(5):e0284824. doi: 10.1371/journal.pone.0284824 (PMC10159165; doi:10.1371/journal.pone.0284824)

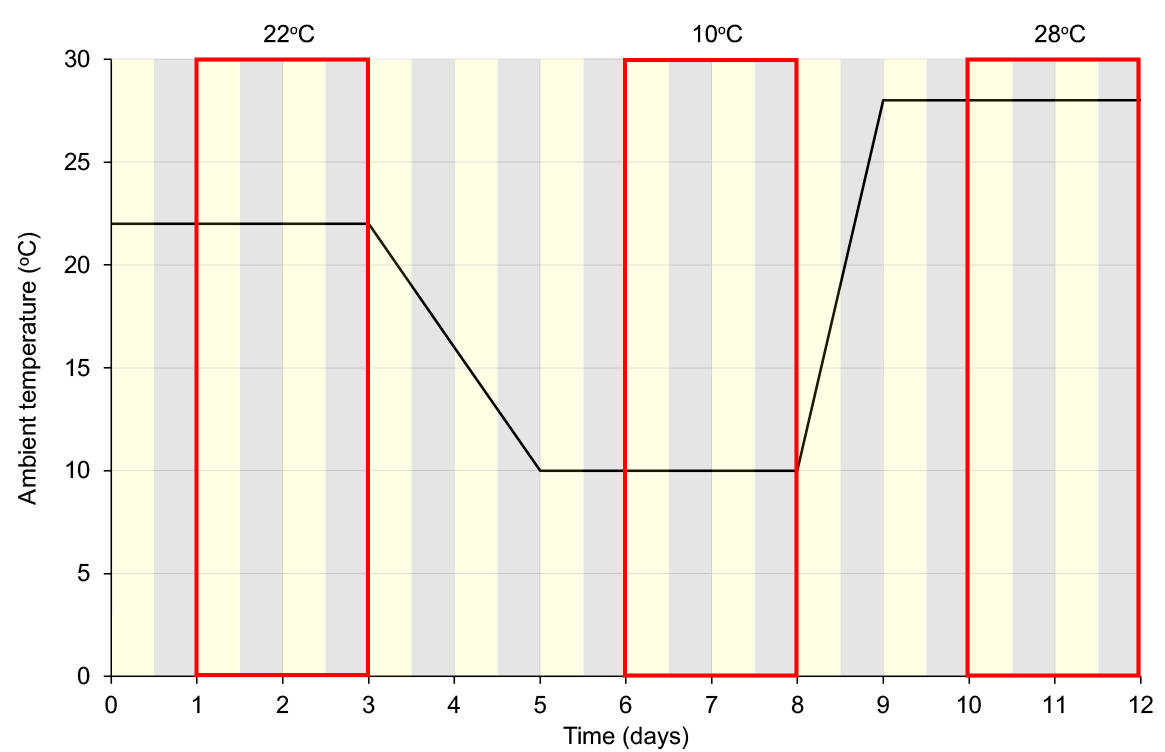

Supplement: S1 Fig — Mice underwent a temperature protocol beginning at standard ambient temperature (22°C) for three days, followed by a two-day ramp to a cold challenge (10°C) for three days, and ending with a one-day ramp to thermoneutrality (28°C) for three days. Each temperature setting included one adaptation day and two days of recorded measurements (red outlines). 48-hour measurements were averaged into 24-hour profiles for statistical analysis. Each day consists of a 12h:12h light:dark cycle (respective yellow and gray shades). (TIF) [file pone.0284824.s001.tif]

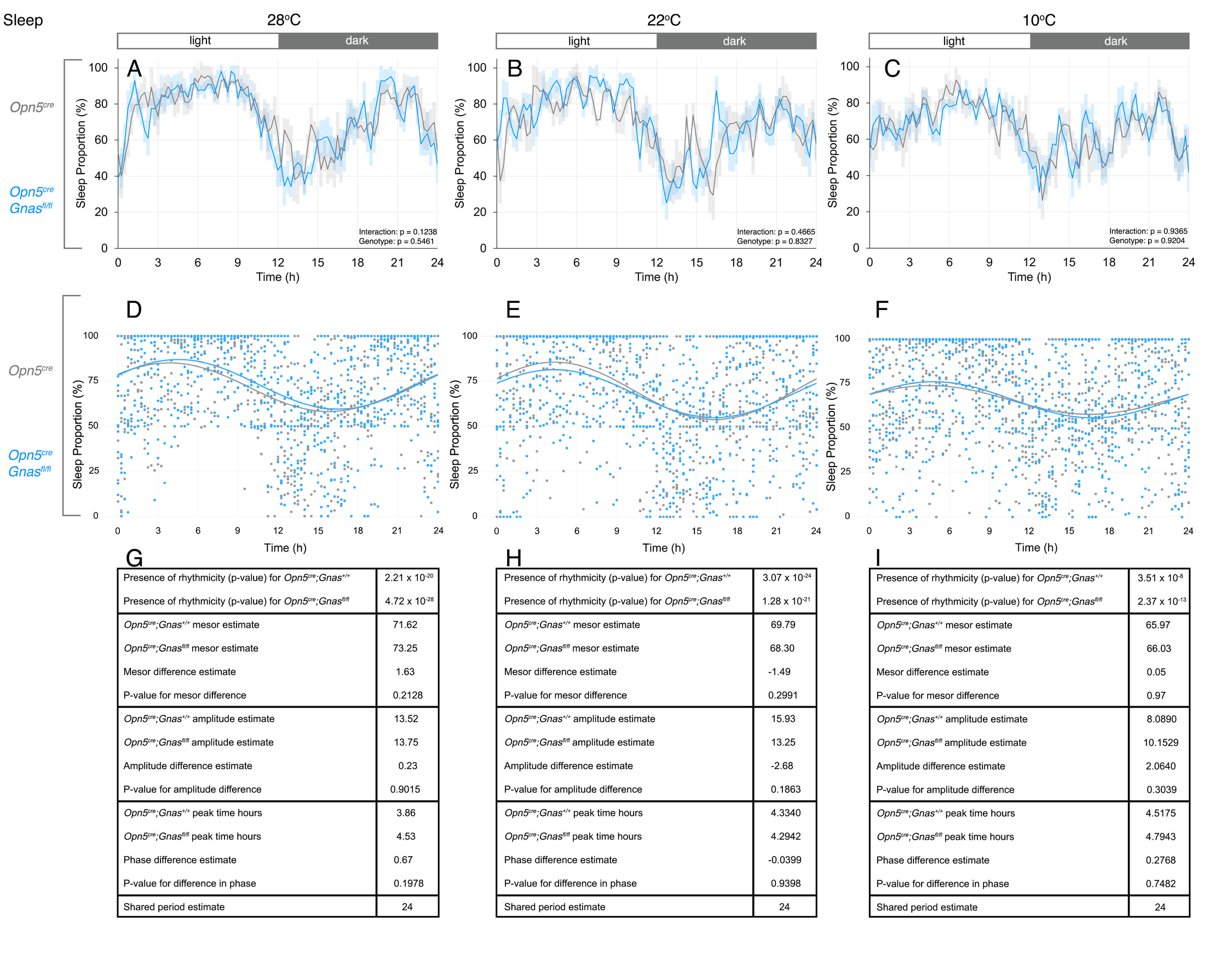

Supplement: S2 Fig — Sleep proportion in Opn5cre;Gnasfl/fl (n = 9, blue) and Opn5cre;Gnas+/+ (n = 7, gray) mice was measured as a percent of each 15-minute interval in which the mouse was immobile after 40 seconds. Respective temperatures are indicated at the top of each column. For time of day on x-axes, t = 0 represents the start of the light phase at 6AM and t = 12 represents the start of the dark phase at 6PM (light:dark bar above each column). 24-hour sleep profiles (A-C) at thermoneutrality (A), standard ambient temperature (B), and during cold-stress (C). Data points in A-C are presented as mean± s.e.m. for 15-minute measurements and analyzed by two-way ANOVA for each 3-hour interval. Significant p-values are indicated above each interval. Cosinor curves (D-F) were generated by CircaCompare for thermoneutrality (D), standard ambient temperature (E), and during cold-stress (F). Estimates of circadian values and p-values (G-I) are listed under each cosinor curve. (TIF) [file pone.0284824.s002.tif]
